# Stimulated Human Mast Cells Secrete Mitochondrial Components That Have Autocrine and Paracrine Inflammatory Actions

**DOI:** 10.1371/journal.pone.0049767

**Published:** 2012-12-17

**Authors:** Bodi Zhang, Shahrzad Asadi, Zuyi Weng, Nikolaos Sismanopoulos, Theoharis C. Theoharides

**Affiliations:** 1 Molecular Immunopharmacology and Drug Discovery Laboratory, Department of Molecular Physiology and Pharmacology, Tufts University School of Medicine, Boston, Massachusetts, United States of America; 2 Department of Biochemistry, Tufts University School of Medicine, Boston, Massachusetts, United States of America; 3 Sackler School of Graduate Biomedical Sciences, Tufts University, Boston, Massachusetts, United States of America; 4 Department of Pharmacy, Tufts Medical Center, Boston, Massachusetts, United States of America; 5 Department of Internal Medicine, Tufts University School of Medicine and Tufts Medical Center, Boston, Massachusetts, United States of America; Centro di Riferimento Oncologico, IRCCS National Cancer Institute, Italy

## Abstract

Mast cells are hematopoietically-derived tissue immune cells that participate in acquired and innate immunity, as well as in inflammation through release of many chemokines and cytokines, especially in response to the pro-inflammatory peptide substance P (SP). Inflammation is critical in the pathogenesis of many diseases, but the trigger(s) is often unknown. We investigated if mast cell stimulation leads to secretion of mitochondrial components and whether these could elicit autocrine and/or paracrine inflammatory effects. Here we show that human LAD2 mast cells stimulated by IgE/anti-IgE or by the SP led to secretion of mitochondrial particles, mitochondrial (mt) mtDNA and ATP without cell death. Mitochondria purified fromLAD2 cells and, when mitochondria added to mast cells trigger degranulation and release of histamine, PGD_2_, IL-8, TNF, and IL-1β. This stimulatory effect is partially inhibited by an ATP receptor antagonist and by DNAse. These results suggest that the mitochondrial protein fraction may also contribute. Purified mitochondria also stimulate IL-8 and vascular endothelial growth factor (VEGF) release from cultured human keratinocytes, and VEGF release from primary human microvascular endothelial cells. In order to investigate if mitochondrial components could be secreted *in vivo*, we injected rats intraperiotoneally (ip) with compound 48/80, which mimicks the action of SP. Peritoneal mast cells degranulated and mitochondrial particles were documented by transimission electron microscopy outside the cells. We also wished to investigate if mitochondrial components secreted locally could reach the systemic circulation. Administration ip of mtDNA isolated from LAD2 cells in rats was detected in their serum within 4 hr, indicating that extravascular mtDNA could enter the systemic circulation. Secretion of mitochondrial components from stimulated live mast cells may act as “autopathogens” contributing to the pathogenesis of inflammatory diseases and may be used as targets for novel treatments.

## Introduction

Mast cells are hematopoietic tissue immune cells that secrete pre-stored mediators, such as histamine and tryptase through degranulation, as well as numerous *de novo* synthesized chemokines and cytokines in response to allergic or non-immune triggers [1], [2]. Interestingly, mast cells are the only cell type that stores pre-formed tumor necrosis factor (TNF) in secretory granules [3]. As a result, mast cells can participate in innate and acquired immunity [4], as well as in inflammation [5], and suspected to participate in the pathogenesis of many diseases including Autism Spectrum Diseases (ASD) [6]. However, the trigger and precise pathogenesis is not known.

We recently showed that human mast cell degranulation and preformed TNF secretion in response to both allergic and non-allergic triggers requires mitochondrial fission and translocation to the cell surface [7]. Mitochondrial shape and localization changes were also shown to occur in T cell activation [8] and chemotaxis [9]. Mitochondria are the primary energy-generating organelles in eukaryotic cells [10], but they also participate in multiple intracellular processes and diseases [11], many of which require mitochondrial fission and translocation [12]. Recent evidence indicates that damage-associated molecular patterns (DAMPs), released from damaged dead cells, can act as “alarmins” [13] and activate polymorphonuclear leukocytes (PMNs) through toll like receptors (TLR)-9, leading to inflammatory responses in the absence of an active infection [14].

Instead, we hypothesized that stimulated live mast cells could secrete mitochondrial components extracellularly, that could further promote inflammation by acting as “auto-pathogens”. We, therefore, investigated if mitochondria could be secreted from stimulated mast cells, in response to allergic and neuropeptide trigger. We now show that human mast cell degranulation triggered by IgE/anti-IgE or substance P (SP) leads to mtDNA secretion without cell death. Mitochondrial components can then stimulate human mast cells, keratinocytes, and primary human microvascular endothelial cells (HMVEC). Human mast cell-derived mtDNA injected ip in rats can be detected in their serum within 4 hr implying that extracellular mitochondrial components can reach distant sites.

## Results

### Mast cell degranulation leads to extracellular mitochondrial particles secretion

**Figure 1:**
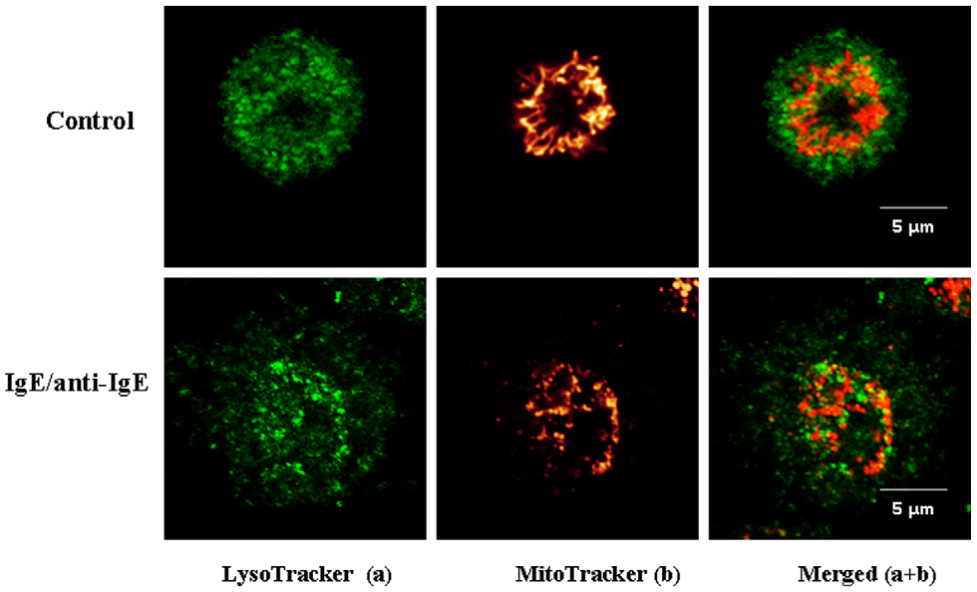
Mast cell degranulation results in extracellular mitochondrial particles translocation. hCBMCs were stained with MitoTracker Deep Red (20 nM) for 20 min and LysoTracker DND green (50 nM) for 30 min, then seeded in glass bottom culture dishes and observed under Leica TCS SP2 Confocal microscope. Mitochondrial distribution was observed in resting (upper panels) and degranulated (bottom panels) mast cells stimulated as shown. The left panels depict secretory granules in green and the middle panels represent mitochondria fluorescence in red. The right panels represent images merged from the two previous panels.

**Figure 2:**
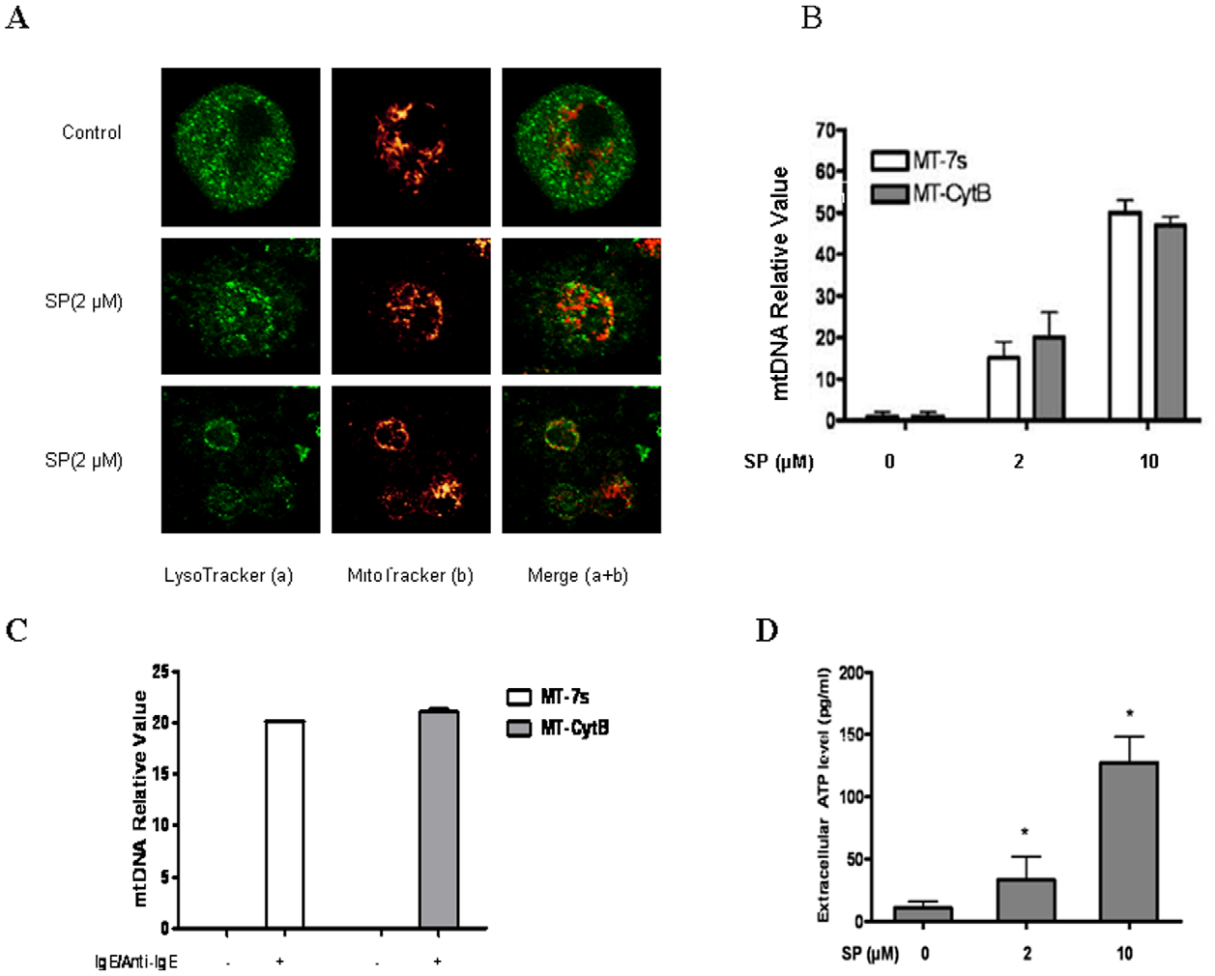
Mast cell degranulation results in extracellular mitochondrial particle secretion. (A) LAD2 cells were stained with MitoTracker and LysoTracker as described in Fig. 1. mitochondria distribution was observed in resting (upper panels) and degranulated (bottom panels) mast cells stimulated as shown. The left panels depict secretory granules in green and the middle panels represent mitochondria fluorescence in red. The right panels represent images merged from the two previous panels. The lower set of three panels show cells in lower magnification as indicated. White rectangles in the middle panels indicate extracellular mitochondriaL particles stained by MitoTracker. Supernatant fluids from both stimulated and control LAD2 cells were collected and assayed for (B, C) mt-7s and mt-CytB, as well as (D) ATP (n = 3; *p<0.05, **p<0.01 compared to control). Sup = Supernatant fluid.

Human cord blood mast cells (hCBMCs) stimulated by IgE/anti-IgE (Fig. 1) for 30 min at 37^°^C undergo rapid degranulation with concomitant mitochondrial fission and translocation from a perinuclear region to a more generalized distribution throughout the cell (Fig. 1), especially close to the cell surface (Fig. 1 upper panels). LAD2 cells stimulated by SP (2 µM) show a similar appearance (Fig. 2A). Surprisingly, many MitoTracker-stained particles are detected outside the cells (Fig. 2A, middle panels, white rectangle), indicating the extracellular presence of functional mitochondria particles, while cell viability remains >99% (Fig. S1). These mitochondria particles are “functional” because they would not otherwise stain with MitoTracker.

### Mast cell degranulation results in extracellular mtDNA release

Supernatant fluid from stimulated LAD2 cells with SP (Fig. 2B) for 30 min or IgE (1 µg/ml) for 2 hr and Anti-IgE (10 µg/ml) for 30 min (Fig. 2C) contains the mtDNA species 7S and Cytochrome B (Cyt B), which are increased 200 times compared with that of control unstimulated cells. In contrast, genomic DNA for GAPDH is not detected (data not shown); indicating the extracellular presence of mtDNA is not due to cell death. Cytochrome C (Cyt C) protein (Fig. S2) in supernatant fluids from stimulated LAD2 cells are also increased, while the cellular Cyt C amount is not changed (Fig. S2), indicating the extracellular Cyt C does not derive from protein increased within cytoplasm that subsequently leaks out. ATP levels are also increased (Fig. 2D). In order to test the percentage of mtDNA released during mast cell degranulation, both mtDNA from supernatant and cells were isolated and the amounts were compared through qPCR. About 7 to 10% of mtDNA was released outside the cells (data not shown). The peptide neurotensin (10 µM) also induced extracellular mitochondria secretion of (data not shown), while mercury chloride (1 µM) used as an environmental trigger had no effect.

### Extracellular release of mtDNA from mast cells is partially stored in exosomes

**Figure 3:**
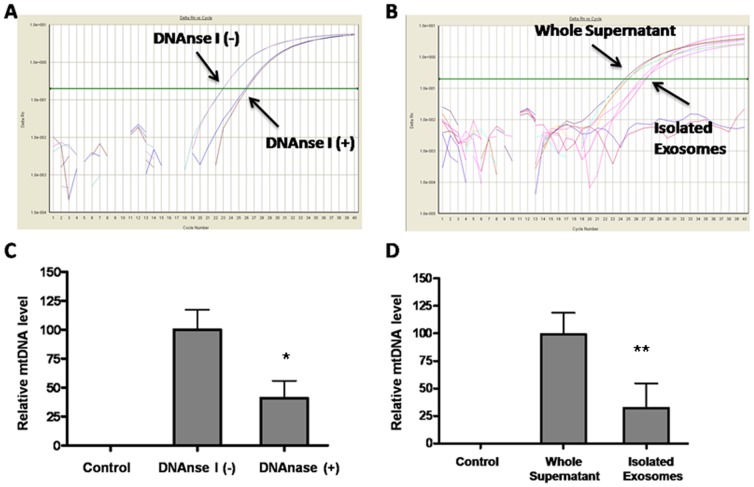
Extracellular release of mtDNA from mast cells is partially stored in exosomes. LAD2 cells were stimulated with SP (2 µM) for 30 min. Supernatant fluids from both stimulated and control LAD2 cells were collected and were treated by DNAase. Quantitative PCR (A) was performed to measure mtDNA level in supernatants with or without DNase treatment (B). Exosomes were isolated from supernatant fluids by Differential Ultracentrifugation followed by (C) mtDNA isolation from exosomes and measured by quantitative PCR. (D) Exosome-containing mtDNA level compared to mtDNA isolated from uncentrifuged supernatant fluids (n = 3; *p<0.05, **p<0.01 compared to control).

In view of previous reports of mast cells secreting active exosomes containing various molecules, we investigated if mtDNA in stimulated mast cells could be secreted inside exosomes. Exosomes were collected by differential centrifugation as previously described. Exosomes are present after mast cell stimulation (Fig. 3B). We then isolated DNA from these exosomes and quantified it by PCR. Exosomes contained mtDNA (Fig. 3B), but not genomic DNA for GAPDH (Data not shown).

To investigate if all of the extracellular mtDNA is confined inside the exosomes rather than absorbed on the pellet during ultracentrifugation, exosomes were treated with DNaseI after resuspension in PBS (Fig. 3A). Such treatment leads to a significant decrease in mtDNA in the DNase-treated as compared to control exosomes (Fig. 3C), indicating that a large portion of extracellular mtDNA (90%) is free. Also, we compared the total amount of extracellular mtDNA and the mtDNA contained in exosomes by quantitative PCR. The results indicate that only about 25% of the mtDNA secreted is contained in exosomes (Fig. 3D).

### Mitochondrial components act as autocrine mast cell triggers of degranulation and inflammasome activation

**Figure 4:**
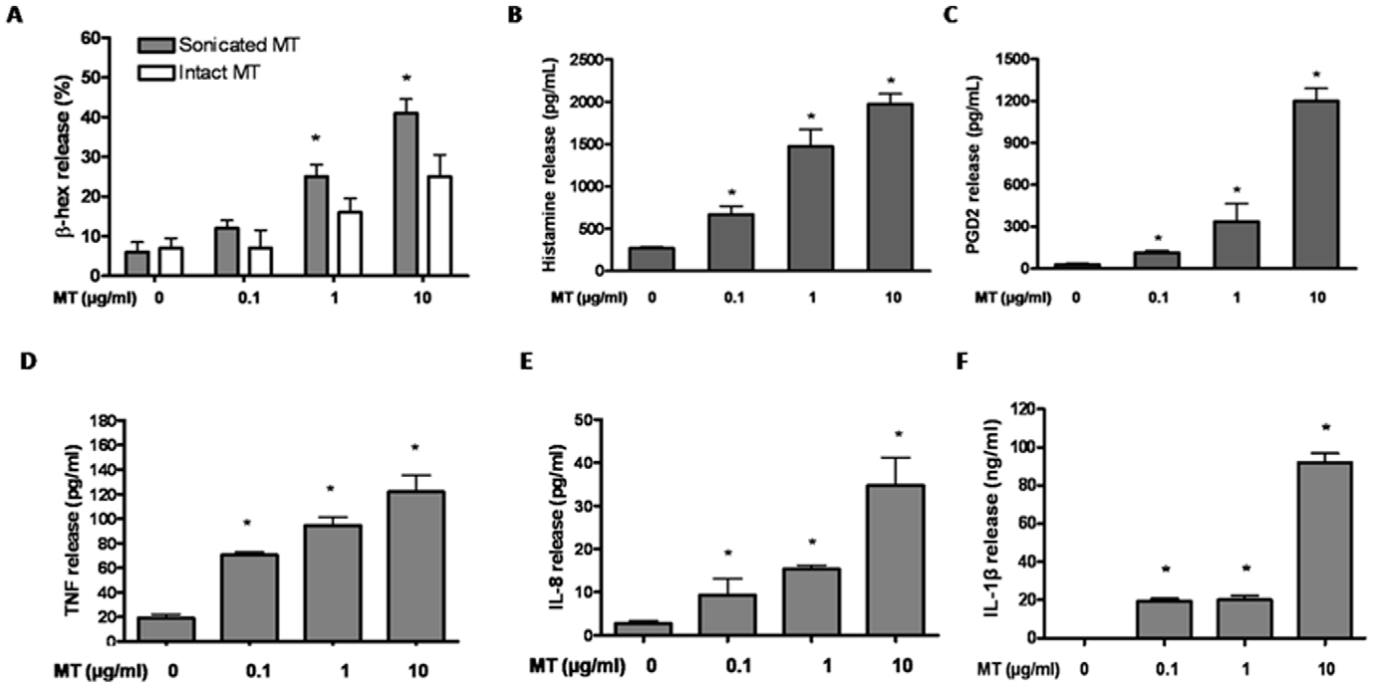
Sonicated mitochondria stimulate human mast cell pro-inflammatory mediators release. LAD2 cells were incubated with mitochondria isolated from mast cells for either 30 min or 24 hr. Supernatant fluids from different conditions were collected. (A) β-hex (B) Histamine and (C) PGD2 release were assayed at 30 min, while (D) TNF, (E) IL-8 and (F) IL-1beta were assayed at 24 hr (n = 3; *p<0.05, compared to control).

It has been reported that mtDNA from shock-damaged rat tissues could stimulate PMN [14]. We, therefore, investigated if secreted mitochondrial components could have functional effects by triggering sterile inflammation. Mitochondria were isolated from LAD2 mast cells, and then either used intact or after sonication to release all inner components. Mitochondria concentrations higher than 0.1 µg/mL triggered LAD2 cell degranulation as shown by β-hexoaminidase (β-hex) release (Fig. 4A), as well as histamine (Fig. 4B) and PGD_2_ secretion (Fig. 4C) in 30 min. Sonicated mitochondria are more potent in triggering degranulation than intact mitochondria (Fig. 4A), suggesting the release of some intra-mitochondrial components. Mitochondria isolated from both LAD2 and sarcoma cells had similar effects (data not shown), indicating their effects are not due to the cell source of mitochondria. The ability of mitochondria to stimulate degranulation could be at least due to the fact that sonicated mitochondria trigger immediate intracellular calcium increase in LAD2 cells (Fig. S3).

LAD2 cells incubated with sonicated mitochondria for 24 hr produced and secreted significant amount of TNF (Fig. 4D) and IL-8 (Fig. 4E). Interestingly, we also detect IL-1beta secretion from mitochondria-stimulated LAD2 cells (Fig. 4F); indicating that mitochondrial components can activate the inflammasome. In order to rule out the possibility that cytokines may derive from mitochondrial particles, we also investigated if IL-8 and TNF are present in isolated mitochondria. However, no cytokine was detected in the mitochondria fraction.

#### Mitochondrial stimulation of mast cells is partially due to activation of P2X7 receptors

**Figure 5:**
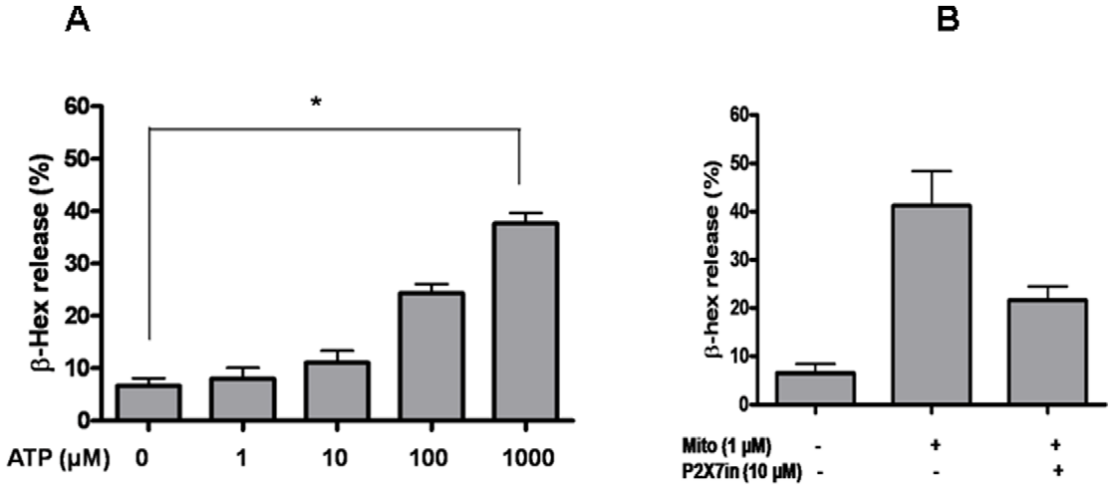
Mitochondrial component stimulation of LAD2 cell β-Hex release is partially P2X7 receptor dependent. LAD2 cells were (A) Stimulated with different concentrations of ATP, or (B) pre-treated with the P2X7 receptor inhibitor for 30 min and then stimulated with mitochondrial components. β-Hex release was measured 30 min later. (n = 3; *p<0.05, compared to control).

We also investigated which components from sonicated mitochondria may activate LAD2 cells. ATP has been reported to stimulate human mast cell degranulation through an IgE-independent mechanism [15]. We show that ATP activated LAD2 cell degranulation (Fig. 5A). Therefore, we tested if inhibition of P2X7 receptor, the primary receptor of ATP, could block this effect. Pre-incubation of LAD2 cells with the P2X7 receptor inhibitor blocked mitochondria-induced degranulation by about 40%, indicating ATP contained in mitochondria is partially responsible for mast cell degranulation (Fig. 5B). We then investigated whether the ability of mitochondrial components to trigger degranulation is also due to liberated fomyl-peptides, which are known to be present in mitochondria. However, we failed to detect degranulation when using isolated fomyl-peptide to stimulate LAD2 cells (Fig. S4). We also purified mitochondrial protein and DNA and tested them separately on LAD2 cells. Most of the stimulatory activity for the TNF release was associated with the protein mitochondrial fraction (Fig. S5).

### Mast cell-derived mitochondrial components have paracrine actions and trigger cytokine release

**Figure 6:**
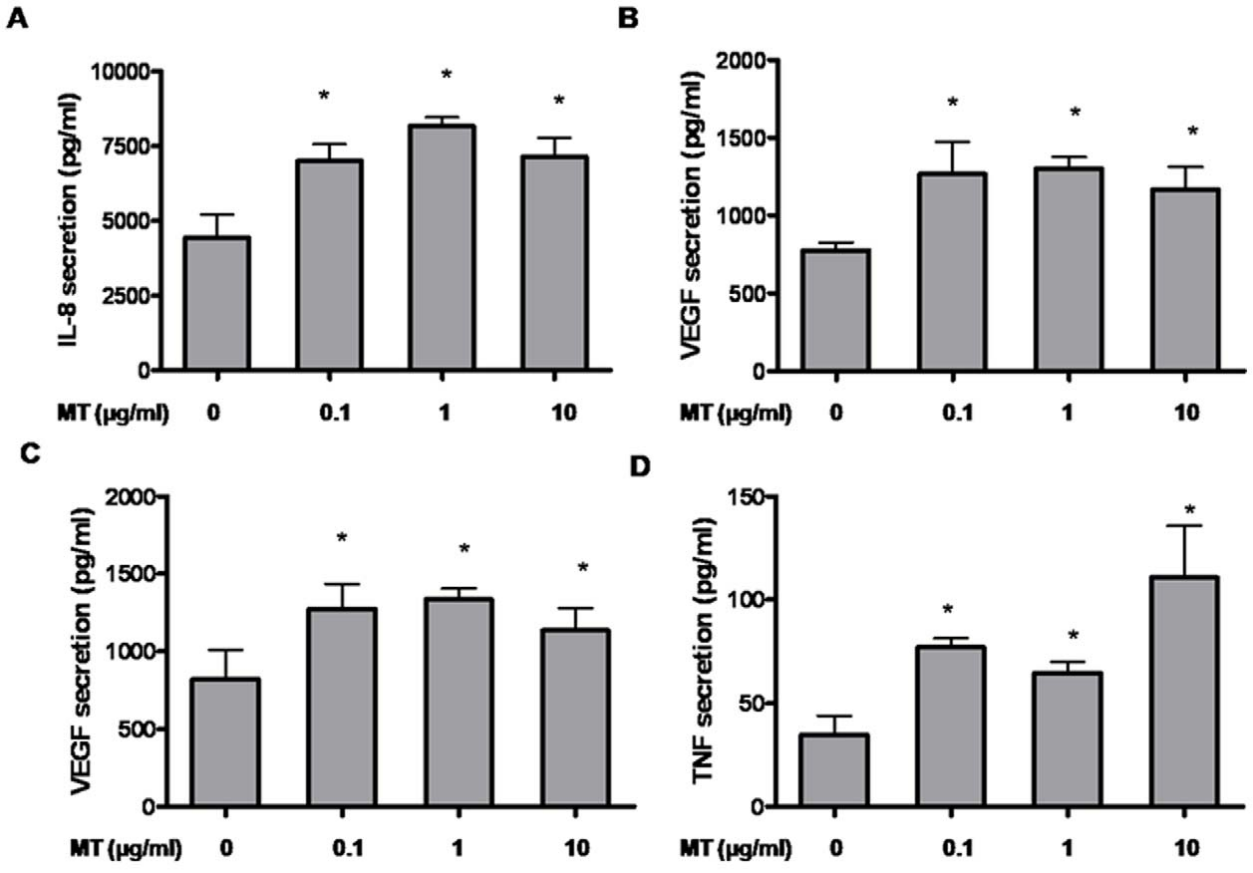
Sonicated mitochondria stimulate human keratinocyte and endothelial cell cytokine release. HaCaT and HMVEC cells were incubated with mitochondria isolated from LAD2 cells for 24 hr. Supernatant fluids from different conditions were collected. Cytokine release from HaCat cells (A) IL-8 and (B) VEGF, as well as from HMVECs (C) VEGF and (D) TNF were measured at 24 hr (n = 3; *p<0.05, compared to control).

Given that mast cells interact with many other cell types especially in the skin, we tested the effect of mast cell-derived mitochondrial components on other key cell types. HaCaT human keratinocytes were incubated with sonicated mitochondria isolated from LAD2 cells for 24 hr. IL-8 (Fig. 6A) and VEGF (Fig. 6B) are significantly elevated in the supernatant fluid following stimulation. Primary human microvascular endothelial cells (HMVEC) stimulated with sonicated mitochondrial components also release significant amounts of VEGF (Fig. 6C) and TNF (Fig. 6D).

### Extracellular mitochondrial components can to enter the systemic circulation

**Figure 7:**
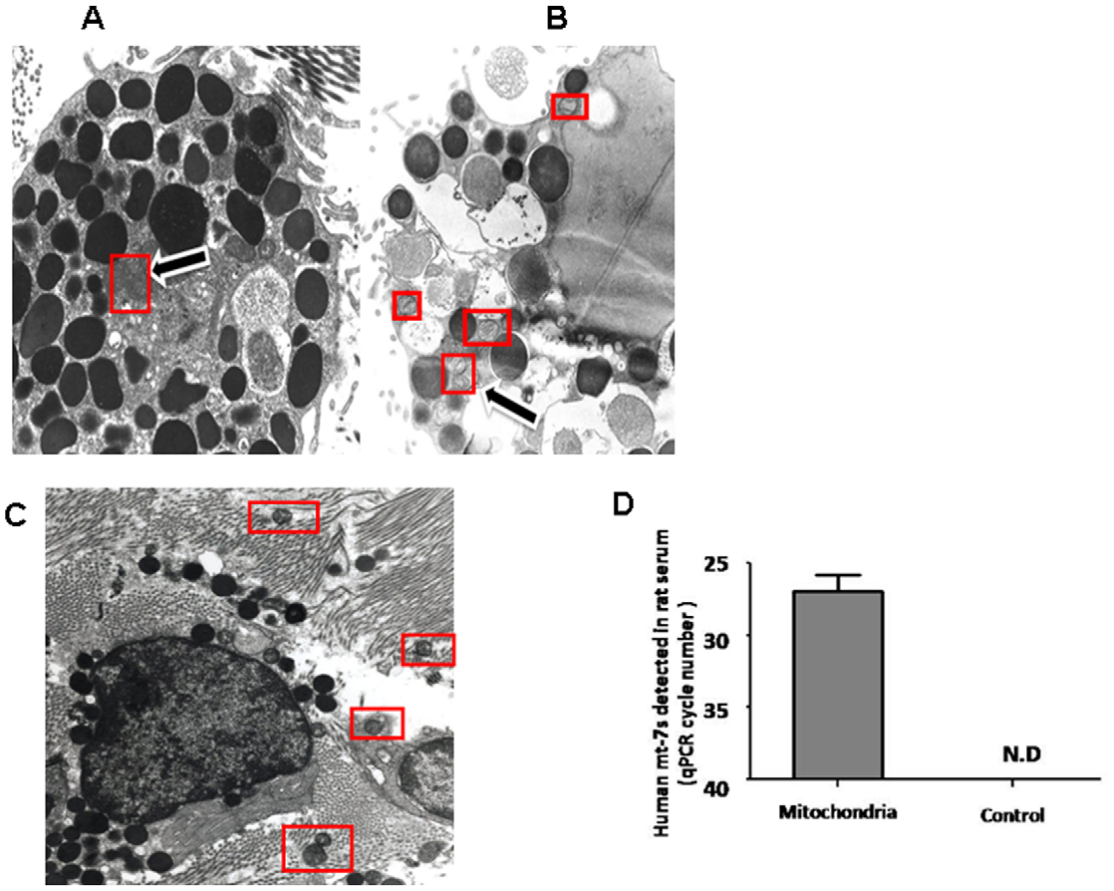
Electron photomicrographs showing mitochondria localization in rat peritoneal mast cells and human mtDNA presence in rat serum. Male rat peritoneal mast cells (A) control with intact electron dense granules and mitochondria inside the cell. (B, C) after C48/80 stimulation showing (B) intense degranulation (*) with most mitochondria at the cell surface close to sites of exocytosis (Magnification: 13,800×), and (C) extracellular mitochondria outside the cell perimeter (Magnification: 4,500×). Mitochondria is shown within red rectangles. (D) Presence of human mitochondria in rat serum following ip injection in male rats (n = 4).

In addition to effects on neighboring cells, it was important to investigate whether mitochondrial components could be secreted *in vivo* and whether they could travel to the systemic circulation. We first injected the mast cell trigger compound 48/80 (1 mg/kg) intraperitoneally into normal male Sprague/Dawley rats. Electron photomicrographs of normal, unstimulated peritoneal mast cells show that mitochondria are found deep inside the normal cells (Fig. 7A). In contrast, smaller mitochondrial particles are mostly present on the cell surface of degranulated mast cells (Fig. 7B). On many occasions, small mitochondrial particles are noted outside the periphery of the activated mast cell (Fig. 7C).

We also isolated mitochondria from human LAD2 cells, and injected them intraperitoneally into normal male Sprague/Dawley rats. Rats were then sacrificed 4 hr later to collect blood. Serum human mt-7s DNAand mt-CytB DNA levels were significantly increased and could have only come from the human mitochondria injected due to PCR primer sequence specificity (Fig. 7D), indicating human mitochondria can enter the systemic circulation.

## Discussion

This is the first time to our knowledge that mitochondrial components are shown to be secreted from activated live cells, and to stimulate mast cells, keratinocytes and endothelial cells to produce pro-inflammtory cytokines. These findings are unlike DAMPs, which are released following major trauma in humans [14] or shock-injured rat tissues [16] that can activate TLR9 receptors on human peripheral polymorphonuclear leukocytes (PMNs) leading to inflammation [14]. In both these cases, the DAMPs came from damaged cells.

Mast cells also express TLRs, including TLR9 that can be activated by bacterial DNA sequences, leading to release of different cytokines [17] that allow mast cells to participate in immunity against bacteria [18]. Mitochondria were bacteria that became symbiotic with eukaryotic cells [19]. mitochondria health is regulated by autophagy that permits generation of functional mitochondria and prevents mitochondria from being released outside the cell [20], [21].

Our results indicate that most of the mast cell-derived mtDNA is free as it is destroyed by DNAase I. Only about 75% of mtDNA appears to be inside exosomes. Mast cells can secrete exosomes [22] and mtDNA was reported inside exosomes secreted from glioblastoma cells [23]. This is the first time to our knowledge that mitochondrial components can stimulate mast cells. At least one of these components is ATP. Extracellular ATP has been shown to trigger and maintain inflammation in asthmatic airways [24]. Moreover, extracellular ATP was recently considered a universal “alarm” signal released from cells under stress and affect neighboring cells [25]. We also showed that mitochondria stimulate keratinocytes and endothelial cells.

Here we also show that peritoneal mitochondrial components can travel to the systemic circulation in rats. The finding is important because it indicates that mtDNA can reach the systemic circulation from distant sites. Our present results could provide a mechanism through which the increased serum mtDNA could contribute to the rare disease mastocytosis [26], [27], which was recently reported to be associated with high serum SP [28], or to autism where we reported increased serum mtDNA in young autistic children as compared to controls [29]. It is interesting that autistic patients exhibit neuro-immune aspects [30], and that children with mastocytosis have a higher risk of developing autism [31]. In fact, mtDNA is neurotoxic in rat brain slices [32]. Moreover, DNA released from dying cells was recently shown to mediate the ability of aluminum to act as adjuvant, having recently replaced thimerosal, in vaccines [33]. Extracellular DNA could, therefore, have neuro-immune actions.

Our findings could not be explained by the leukemic nature of LAD2 mast cells because hCBMC behave similarly. One obvious question is whether mast cells are a reasonable source to explain the presence of mtDNA in the serum since mast cells do not circulate. A rough calculation indicates that mast cells secrete about 5% of their mtDNA during degranulation, Given that mast cells secrete potent vasodilatory molecules and we show that mitochondrial components stimulate endothelial cells, some mtDNA could enter the circulation. Another possibility would be that some mtDNA is secreted from circulating basophils, which can participate together with mast cells in innate immunity [34].

In conclusion, the present findings point to a unique and heretofore unrecognized function of mitochondrial components secreted from live activated mast cells with autocrine and paracrine pro-inflammatory effects. Extracellular mitochondrial components could act as “autopathogens” and be the “missing trigger” in certain auto-immune and auto-inflammatory diseases. Detecting circulating mtDNA could be used for diagnosis, while neutralizing extracellular mtDNA sequencesorother mitochondrial components may be used as novel therapeutic approaches.

## Materials and Methods

### Ethics Statement

LAD2 cells (kindly provided by Dr. A.S. Kirshenbaum, National Institutes of Health, NIH) were derived from a single patient with human mast cell leukemia as reported previously and also by us in Plos One [35], [36]. hCBMCs were cultured as described below. Human umbilical cord blood was collected following normal uncomplicated deliveries at Tufts Medical Center (Boston, MA) approved by Tufts Medical Center Human Institutional Review Board (IRB). No consent form is required for discarded tissue without identifiers (Exemption #4).

Rats were injected as described in the methods under a protocol approved by Tufts University's Institution Animal Care and committee (IACUC).

### Materials

DNAase I, CPG-DNP, protease inhibitor cocktail, P2X7 receptor antagonist, TNF and DMSO were purchased from Sigma-Aldrich (St Louis, MO). Mitotracker Deep Red and LysoTracker DND were purchased from Molecular Probes (Eugene, OR). IL-8, TNF and VEGF ELISA kits were purchased from R&D Systems (Minneapolis, MN). Mt-7s and mt-CytB Taqman probes and Taqman Master Mix were purchased from Applied Biosystems (Foster City, CA). MDIVI-1 was kindly supplied by Dr. O. Shirihai (Boston University, Boston, MA).

### Culture of human mast cells, keratinocytes and primary human microvascular endothelial cells

LAD2 cells were cultured in StemPro®-34 SFM medium (Invitrogen, Carlsbad, CA) supplemented with 100 U/mL penicillin/streptomycin and 100 ng/mL recombinant human stem cell factor (rhSCF, kindly supplied by Sweden Orphan Biovitrum AB, Stockholm, Sweden). These cells have been used numerous times in our laboratory [37].

Hematopoietic stem cells (CD34^+^) were isolated by positive selection of CD34^+^/AC133^+^ cells with magnetic cell sorting using an AC133^+^ cell isolation kit (Miltenyi Biotec, Auburn, CA) as previously reported [38]. CD34^+^ cells were suspended in AIM-V Medium (Gibco-BRL, Carlsbad, CA), supplemented with 100 ng/mL recombinant human stem cell factor (rhSCF; kindly supplied by Biovitrum, Stockholm, Sweden) and 50 ng/mL IL-6 (Millipore, Billerica, MA) for 12 to 16 weeks. The purity of hCBMCs was evaluated by immunocytochemical staining for tryptase as previously described [38]. Mast cells (100% purity) cultured over 12 weeks were used for the experiments. HaCaT human keratinocytes (kindly supplied by Dr. A. Slominsky, University of Tennessee, Memphis, TN) were cultured in DMEM medium (Invitrogen) with 10% FBS. Primary human microvascular endothelial cells (HMVEC) were kindly donated by Dr. Ira Herman (Tufts University, Boston, MA) and were cultured in RPMI 1640 medium with 5% FBS.

### Mast cell stimulation

hCBMCs and LAD2 cells were stimulated by IgE/Anti-IgE as described previously [39]. Briefly, 1 µg/ml IgE (Millipore, Billerica, MA) was used for 2 hr, cells were washed and then stimulated with 10 µg/ml anti-IgE (DAKO, Carpinteria, CA) for 30 min. LAD2 cells were stimulated by SP (2 µM) for either 30 min or 24 hr.

### Confocal microscopy

Functional mitochondria were stained with MitoTracker, which becomes fluorescent only when it is oxidized inside intact mitochondria. Secretory granules were stained with LysoTracker because the average pH of mast cell granules is about 5.5, similar to that of lysosomes [40]. LAD2 cells were incubated with 20 nM MitoTracker Deep Red Probe (Invitrogen) for 20 min and 50 nM LysoTracker DND probe for 30 min. Cells were washed, moved to glass bottom culture dishes (MatTek, Ashland, MA) and observed using a Leica TCS SP2 Confocal microscope (Leica, Japan). The percentages of cells with mitochondria redistribution were counted from 100 randomly selected mast cells in each experiment. Confocal digital images were processed using the National Institute of Health ImageJ 1.32 and Adobe Photoshop 7.0 Programs.

### Mitochondria isolation

The Mitochondria Isolation Kit for cells (Pierce Scientific, Rockford, IL) was used to isolate mitochondria from cultured cell lines. Mitochondria were isolated under sterile conditions at 4°C following the directions of the manufacturer. The specificity of the mitochondria isolation kit is provided by the manufacturer and excludes lysosomes, nucleus and secretory granules (Qiagen, CA). There was no LDH detected in the supernatant fluids of activated mast cells, indicating no cytosolic component contamination. Mitochondria were then sonicated for 2 min at 4°C to release all inner components. Mitochondrial DNA and protein concentration were determined by NanoDrop 2000 supplied by Thermo Scientific (Waltham MA).

### Preparation of mtDNA

Isolated mitochondria pellets from human LAD2 mast cells were suspended in 1 mL of PBS. Mitochondrial DNA was extracted from the isolated mitochondria of LAD2 cells under sterile conditions using DNeasy Blood & Tissue kit from Qiagen (Valencia, CA). No protein contamination was found in mtDNA and nuclear DNA GAPDH was not detectable in 45 cycles by quantitative PCR.

### β–Hexosaminidase, histamine and PGD_2_ release assay

Secretion of β-hexosaminidase (β-hex), as an index of mast cell degranulation, was detected using a fluorometric assay as previously reported. Briefly, β-hex activity in the supernatant fluid and cell lysates (0.5×10^5^ cells, lysed with 1% Triton X-100 to measure residual cell-associated β-hex) were assayed by incubation with substrate solution (p-nitrophenyl-N-acetyl-β-D-glucosaminide from Sigma) in 0.1 M NaOH/0.2 M glycine. Absorbance was measured at 405 nm in a plate reader, and the results are expressed as the percentage of β-hex released over the total. Mast cell histamine and PGD_2_ release was measured from supernatants fluids using ELISA kits (R&D Systems) according to the manufacturer's instructions.

### Cytokine release assay

hCBMCs, LAD2, HaCaT and HMVEC cells were treated with the indicated amounts of mitochondria for 24 hrs. IL-1 beta, IL-8, TNF and VEGF levels into the supernatant fluids were measured by ELISA.

### Cytosolic Calcium Measurements

Cytosolic calcium was measured in LAD2 cells at 37°C using Fura-2 (Invitrogen) as indicator. LAD2 cells suspended in Tyrode buffer, were loaded with 30 nM Fura-2 AM for 20 min to allow Fura-2 to enter the cells. After centrifugation to remove excess dye, cells were resuspended in Tyrode buffer at a concentration of 10^6^/ml and incubated for another 20 min. Cells were then transferred to 96-well plates (100 µl/well) and SP (2 µM) was added. Real-time Fura-2 fluorescence was read by MDC FlexStation II (Molecular Devices, Sunnyvale, CA, USA) at an excitation wavelength of 340 nm/380 nm and emission wavelength of 510 nm. Results were analyzed according to the Invitrogen Fura-2 protocol and reported as the relative value of OD 340/380 nm as described previously [41].

### Cell viability test

Cell viability was determined by Evans blue staining along with LDH measurement in the supernatant fluid.

### Quantitative PCR

Total DNA from mast cell supernatant fluids was isolated using DNA Mini Kit (Qiagen) according to the manufacturer's instructions. In order to measure mt-7s and mt-CytB release, quantitative real time PCR was performed using Taqman gene expression assays using the following probes: Hs 02596861_s1 for mt-7s and Hs 02596867_s1 for mt-CytB (Applied Biosystems). The cycling conditions consisted of 1 cycle of 50°C for 2 min, 1 cycle of 95°C for 10 min, followed by 40 cycles of 95°C for 15 s and 60°C for 1 min. GAPDH was used as internal control to test for genomic DNA contamination. Samples that produced no PCR products after 45 cycles were considered ‘undetectable’ and the Ct number was set to 40 for statistical purposes. The human mtDNA probes did not detect any rat mtDNA up to 50 cycles, indicating no cross-reaction between human probes and rat mtDNA.

### Intraperitoneal administration of C48/80 and mitochondria

Male Sprague/Dawley rats (280±20 g, Charles River, Wilmington, MA) were injected ip with compound 48/80 (C48/80) at 1 mg/kg in 0.2 ml normal saline. Other rats were injected ip with mitochondria (0.01 mg/ml in 0.2 ml normal saline) prepared under sterile conditions from LAD2 cells as described above. Control rats were injected with normal saline. Four hr after treatments, rats were sacrificed under isofluorane anesthesia and blood was obtained from neck veins following decapitation. The peritoneal cavity was opened and fixed *in situ* with 0.4% modified Karnovsky's fixative containing 0.2% paraformaldehyde, 3% glutaraldehyde, and 0.5% tannic acid in 0.1 mM Na-cacodylate buffer. Serum mtDNA levels were detected by qPCR as described above.

### Transmission electron microscopy

Rat peritoneal samples from control (n = 3) and stimulated (n = 3) rats were fixed in modified Karnovsky's fixative containing 0.2% paraformaldehyde, 3% glutaraldehyde, and 0.5% tannic acid in 0.1 mM Na-cacodylate buffer. Sections (5 µm) were cut using a microtome and observed using a Philips-300 TEM. High quality glossy photographs of individual mast cells were evaluated by three independent operators for the number of mitochondria and their distance from the cell surface. All mast cells from rats treated with C48/80 showed evidence of degranulation to different extent defined as 5 or more altered secretory granules.

### Data Analysis

Image analysis was performed with the experimental conditions blinded. For each experimental condition, 20 or 30 confocal images were randomly taken from different areas of the microscopic field. ImageJ software (NIH) was used for image processing. All data are expressed as mean±SD as indicated in the figure legends. Statistical significance between experimental samples and controls was determined by the Student's *t*-test. When more than one sample was compared to the controls, ANOVA was used followed by Dunnett's test for multiple comparisons.

Significant differences were considered if p<0.05.

## Supporting Information

Figure S1 **LAD2 cells were stimulated with SP (2 µM for 24 hr) and cell viability was determined by Trypan blue exclusion assay.**
(TIF)

Figure S2 CytochromeC (Cyt C) protein in supernatant fluids from LAD2 cells stimulated by SP compared to cellular CytC amount levels (n = 3; *p<0.05, compared to control). Sup = Supernatant fluid.
(TIF)

Figure S3 **LAD2 cells were first stained with Fura-2, washed and then stimulated with different mitochondria concentrations as shown.** The experiments were repeated three times and the figure shown is representative of three similar results. (n = 3; *p<0.05, compared to control).
(TIF)

Figure S4 **Different concentration of N-Fomyl-peptide and CpG-ODN was used to stimulated LAD2 cells.** Beta-hex release was measured 30 mins after stimulation. (n = 3; *p<0.05, compared to control).
(TIF)

Figure S5 hCBMCs were incubated with either sonicated mitochondria, mitochondrial protein, or mtDNA at the concentrations shown (n = 3; *p<0.05, compared to control).
(TIF)
