# Supplementary figures and images for: Stimulated Human Mast Cells Secrete Mitochondrial Components That Have Autocrine and Paracrine Inflammatory Actions
Source: PLoS One. 2012 Dec 17;7(12):e49767. doi: 10.1371/journal.pone.0049767 (PMC3524249; doi:10.1371/journal.pone.0049767)

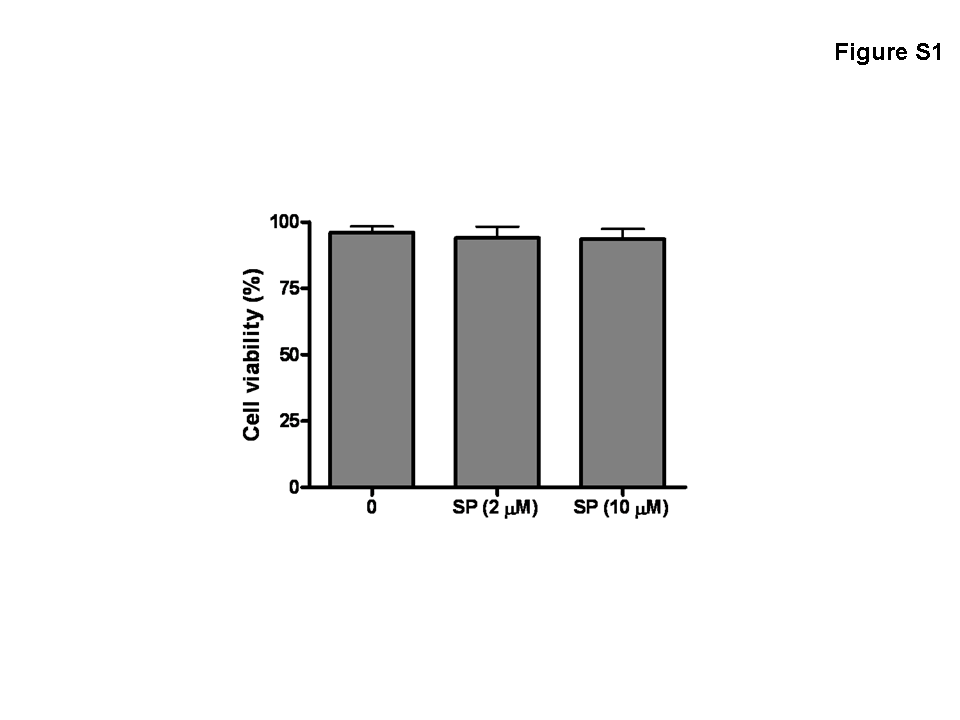

Supplement: Figure S1 — LAD2 cells were stimulated with SP (2 µM for 24 hr) and cell viability was determined by Trypan blue exclusion assay. (TIF) [file pone.0049767.s001.tif]

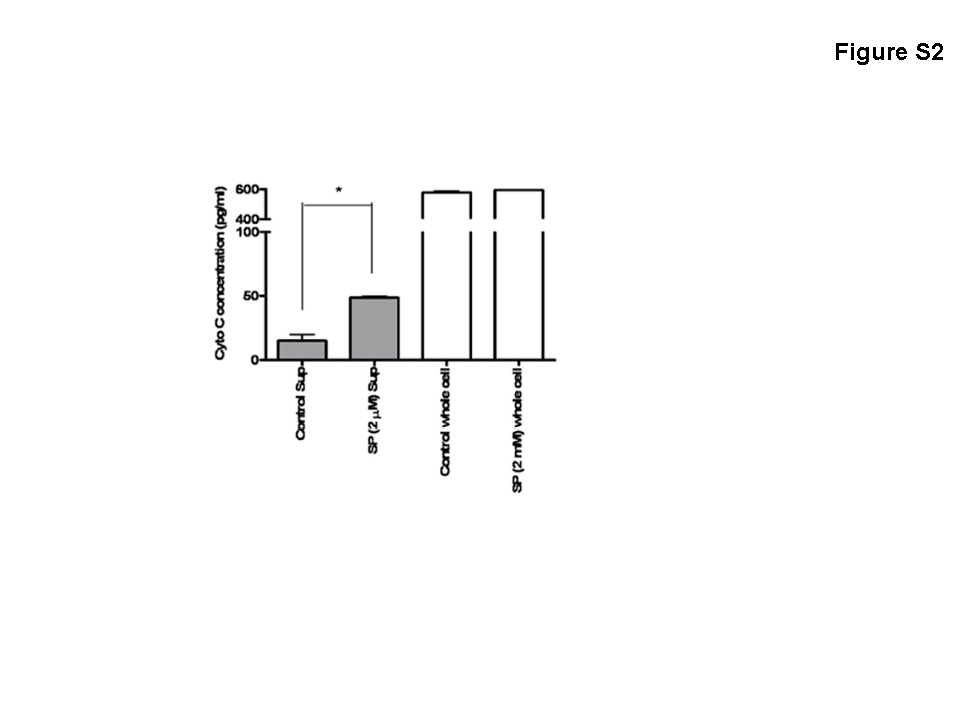

Supplement: Figure S2 — CytochromeC (Cyt C) protein in supernatant fluids from LAD2 cells stimulated by SP compared to cellular CytC amount levels (n = 3; *p<0.05, compared to control). Sup = Supernatant fluid. (TIF) [file pone.0049767.s002.tif]

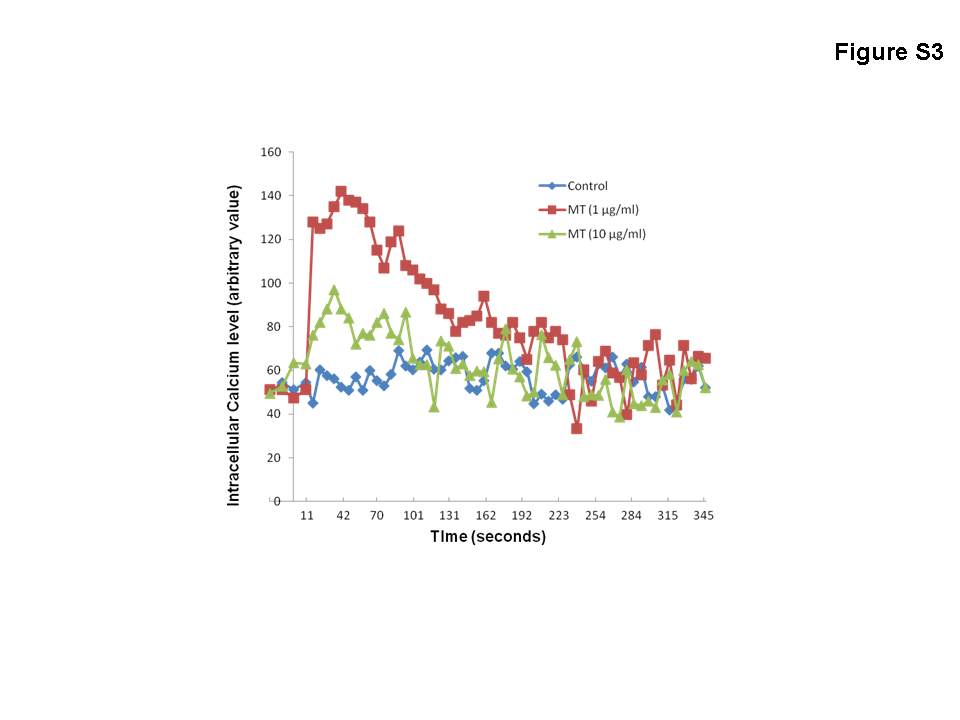

Supplement: Figure S3 — LAD2 cells were first stained with Fura-2, washed and then stimulated with different mitochondria concentrations as shown. The experiments were repeated three times and the figure shown is representative of three similar results. (n = 3; *p<0.05, compared to control). (TIF) [file pone.0049767.s003.tif]

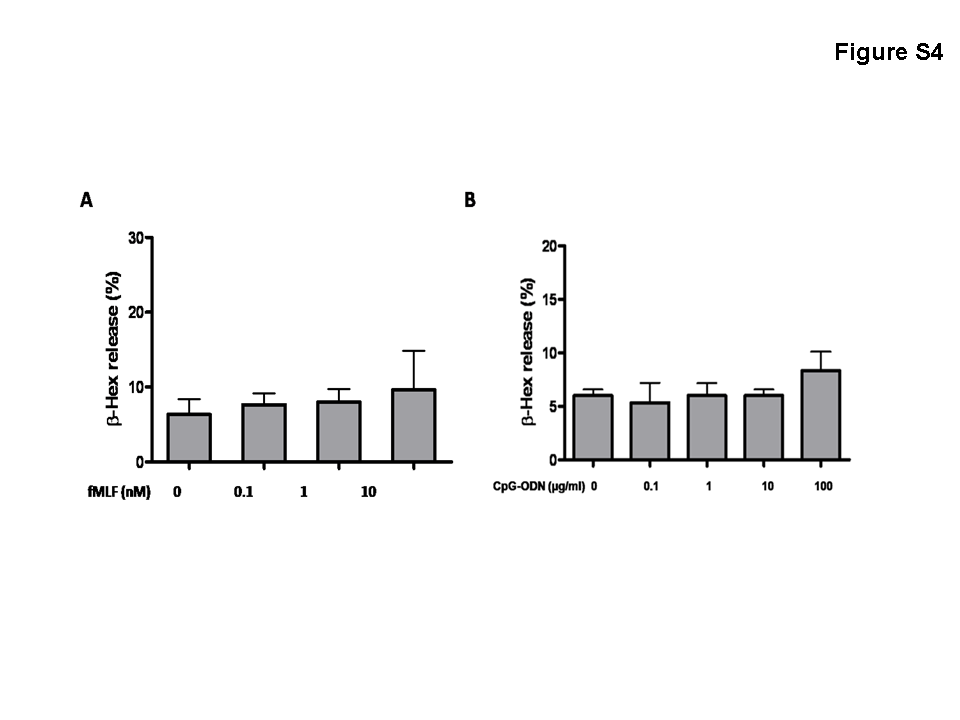

Supplement: Figure S4 — Different concentration of N-Fomyl-peptide and CpG-ODN was used to stimulated LAD2 cells. Beta-hex release was measured 30 mins after stimulation. (n = 3; *p<0.05, compared to control). (TIF) [file pone.0049767.s004.tif]

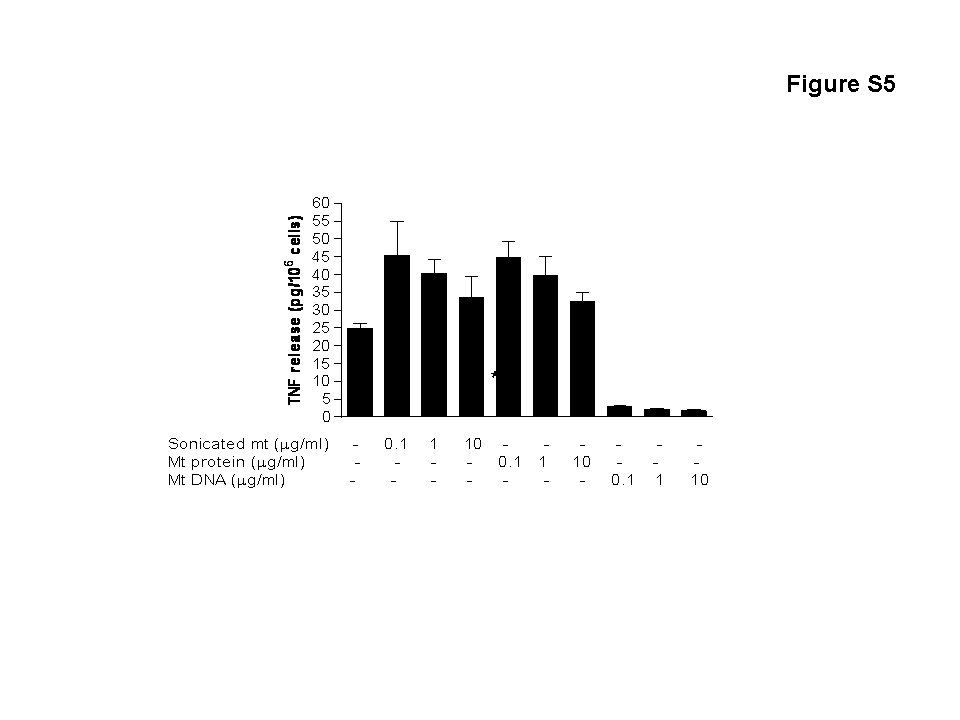

Supplement: Figure S5 — hCBMCs were incubated with either sonicated mitochondria, mitochondrial protein, or mtDNA at the concentrations shown (n = 3; *p<0.05, compared to control). (TIF) [file pone.0049767.s005.tif]
